# ANKRD44 Gene Silencing: A Putative Role in Trastuzumab Resistance in Her2-Like Breast Cancer

**DOI:** 10.3389/fonc.2019.00547

**Published:** 2019-06-26

**Authors:** Marco La Ferla, Francesca Lessi, Paolo Aretini, Davide Pellegrini, Sara Franceschi, Elena Tantillo, Michele Menicagli, Ivo Marchetti, Claudia Scopelliti, Prospero Civita, Claudia De Angelis, Lucrezia Diodati, Ilaria Bertolini, Manuela Roncella, Liam A. McDonnell, Jacob Hochman, Marzia Del Re, Cristian Scatena, Antonio G. Naccarato, Andrea Fontana, Chiara M. Mazzanti

**Affiliations:** ^1^Fondazione Pisana per la Scienza - Genomic Section, Pisa, Italy; ^2^Fondazione Pisana per la Scienza - Proteomic Section, Pisa, Italy; ^3^NEST, Scuola Normale Superiore, Pisa, Italy; ^4^Scuola Normale Superiore, Pisa, Italy; ^5^Cytopathology Section, Azienda Ospedaliero-Universitaria Pisana (AOUP), Pisa, Italy; ^6^Medical Oncology Unit, Azienda Ospedaliero-Universitaria Pisana (AOUP), Pisa, Italy; ^7^Breast Cancer Center, Azienda Ospedaliero-Universitaria Pisana (AOUP), Pisa, Italy; ^8^Department of Cell and Developmental Biology, the Hebrew University of Jerusalem, Jerusalem, Israel; ^9^Unit of Clinical Pharmacology and Pharmacogenetics, Department of Clinical and Experimental Medicine, University of Pisa, Pisa, Italy; ^10^Department of Translational Research and New Technologies in Medicine and Surgery, University Hospital of Pisa, Pisa, Italy

**Keywords:** Her2+ breast cancer, Trastuzumab resistance, ANKRD44, next generation sequencing, LC-MS/MS, gene silencing

## Abstract

Trastuzumab is an effective therapeutic treatment for Her2-like breast cancer; despite this most of these tumors develop resistance to therapy due to specific gene mutations or alterations in gene expression. Understanding the mechanisms of resistance to Trastuzumab could be a useful tool in order to identify combinations of drugs that elude resistance and allow a better response for the treated patients. Twelve primary biopsies of Her2+/hormone receptor negative (ER-/PgR-) breast cancer patients were selected based on the specific response to neoadjuvant therapy with Trastuzumab and their whole exome was sequenced leading to the identification of 18 informative gene mutations that discriminate patients selectively based on response to treatment. Among these genes, we focused on the study of the ANKRD44 gene to understand its role in the mechanism of resistance to Trastuzumab. The ANKRD44 gene was silenced in Her2-like breast cancer cell line (BT474), obtaining a partially Trastuzumab-resistant breast cancer cell line that constitutively activates the NF-kb protein via the TAK1/AKT pathway. Following this activation an increase in the level of glycolysis in resistant cells is promoted, also confirmed by the up-regulation of the LDHB protein and by an increased TROP2 protein expression, found generally associated with aggressive tumors. These results allow us to consider the ANKRD44 gene as a potential gene involved in Trastuzumab resistance.

## Introduction

Her2 is a transmembrane receptor with tyrosine kinase activity. This protein belongs to the EGFR family which is composed of four receptors: Her1/EGFR, Her2, Her3, and Her4. EGFR proteins are involved in many biological processes, such as proliferation, differentiation, cell motility and apoptosis (1), through different signal transduction pathways (2, 3) including the PI3K/Akt and the Ras/Raf/MEK/MAPK pathway (4). In physiological condition a specific level of Her2 is required for breast development (5), but its amplification and overexpression causes the dysregulation of normal cellular control and the formation of aggressive breast tumor cells (1, 6). Genetic alterations in Her2 gene are found in almost the 30% of breast cancer (BC) patients and are associated with a poor prognosis (7), causing an increased protein expression on the tumor cell surface which leads to an increased cell proliferation, cell survival (8) and tumoral resistance to anticancer therapies.

The monoclonal antibody Trastuzumab (TRA) is currently the backbone of treatment both in the adjuvant and in the advanced setting for patients with Her2-like BC. The mechanisms, by which TRA inhibits Her2-overexpressing cancer cells growth, are not completely defined, but down-modulation of PI3K/Akt and/or Ras/MAPK signaling pathways are essential features of TRA response leading to eventual cell cycle arrest (9). However, nearly 25% of patients present disease progression at 10 years after TRA treatment, in the adjuvant setting (10) and only 11–15% show a partial remission when TRA has been administrated as monotherapy (11, 12). These results indicate that most of the patients become resistant to TRA, although their initial response. Several mechanisms of intrinsic and acquired resistance have been proposed, including disruption of receptor-antibody interaction (13, 14), compensatory signaling by other Her family receptors (15), loss of PTEN and mutation of PIK3A (16, 17), signaling by the insulin-like growth factor I receptor (18, 19).

In recent years, other potential mechanisms of acquired resistance to TRA in Her2-like BC have been discovered and studied, such as the increased phosphorylation of the nuclear factor kappa B (NF-kB) p65 subunit (20, 21). Understanding the molecular mechanisms that contribute to the acquired resistance will ultimately allow for the identification of biomarkers that can be used to predict response to TRA therapy, as well as the identification of molecular targets for new therapeutics development.

In this study, we performed a whole-exome analysis of 12 formalin-fixed, paraffin-embedded (FFPE) biopsies of Her2-like BC patients composed of Full Responders (FR) and Partial Responders (PR) to TRA neoadjuvant therapy. We identified a list of 18 genes whose mutational profile discriminates selectively the two groups of patients. Among these genes we focused our attention on the ANKRD44 gene, highly mutated in PR patients and in particular on a single nucleotide polymorphism (SNP) found in the PR patients. ANKRD44 gene encodes for an ankyrin repeat domain protein, member of a putative regulatory ARS subunit of the protein phosphatase 6 (PP6) complex (22, 23).

It is well-known that the ARS subunit plays a central role in the PP6 activity by recognizing phosphoprotein substrate (24). The ANKRD44 SNP identified by our analysis has an high probability to be deleterious for the protein conformation. Therefore, we performed ANKRD44 gene expression silencing in human Her2-like BC cell line (BT474) to investigate its involvement in TRA resistance.

Our novel findings have important implications for the future development of therapeutics for Her2-like and TRA-resistant cancers.

## Materials and Methods

### Patient Samples

A total of 12 patients with stage III Her2-positive/hormone receptor-negative (ER-/PgR-) BC were treated with epirubicin (90 mg/sqm) plus cyclophosphamide (600 mg/sqm) every 3 weeks for 4 cycles followed by paclitaxel (80 mg/sqm, weekly for 12 weeks) and TRA (loading dose of 8 mg/kg, then at 6 mg/kg every 3 weeks for 18 cycles started in combination with paclitaxel). Definitive surgical intervention was carried out within 4 weeks of the final dose of paclitaxel. As mentioned above, the patients were classified on the basis of the response to treatment: 6 FR patients and 6 PR patients (Table 1). The FR patients presented a pathological complete response (pCR) to the therapy with a definitive dissolvence of the infiltrating tumor both in the breast and in the axilla (i.e., ypT0/is ypN0). The PR patients manifested an incomplete pCR with a neoplastic residue >10% after the treatment. We collected FFPE tumor biopsies for FR patients and for PR patients upon surgical removal after pathologist's review (Department of Pathology, University of Pisa, Italy) prior to chemotherapy. Patients who did not completely respond to the therapy, went into surgery subsequently and the FFPE primary tumors, post therapy were collected for the analysis.

**Table 1:** Clinical samples: clinical and pathological informations for the 12 patients under study (6 full responders and 6 partial responders).

**Sample**	**Age**	**Biopsy**	**cTNM**	**Stage**	**ER**	**PgR**	**HER-2 (IHC)**	**Surgery**	**Histology**	**yTNM**	**Response**
FR1	66	Ductal infiltrating carcinoma	cT4N1	IIIB	0	<1	3+	Right superior-external quadrantectomy and axillar lymphadenectomy	Absence of neoplastic residue	yT0N0	pCR
FR2	50	Infiltrating carcinoma	cT3N3	IIIC	0	<1	3+	Right superior-external quadrantectomy and axillar lymphadenectomy	Microfoci of DCIS	yTisN0	pCR
FR3	41	Infiltrating carcinoma	cT4N2	IIIB	<1	<3	3+	Right radical mastectomy Madden	Microfoci of DCIS	yTisN0	pCR
FR4	71	Ductal infiltrating carcinoma	cT3N+	IIIA	0	0	3+	Right superior QUART and lymphadenectomy	Absence of neoplastic residue	yT0N0	pCR
FR5	56	Infiltrating carcinoma	cT2N0	IIA	1	3	3+	Left radical mastectomy and lymphadenectomy	Absence of neoplastic residue	yT0N0	pCR
FR6	77	Ductal infiltrating carcinoma	cT2N3	IIIC	0	0	3+	Left radical mastectomy and lymphadenectomy	Absence of neoplastic residue	yT0N0	pCR
PR1	43	Ductal infiltrating carcinoma	cT4N1	IIIB	0	0	2+	Left radical mastectomy	Ductal infiltrating carcinoma	yT2N0	PR
PR2	44	Ductal infiltrating carcinoma	cT3N1	IIIA	0	2	3+	Left external quadrantectomy and lympadenectomy	Ductal infiltrating carcinoma	yT1bN0	PR
PR3	58	Ductal infiltrating and *in situ* carcinoma	cT4N1	IIIB	<1	<1	3+	Right radical mastectomy Madden	Foci of ductal infiltrating carcinoma	yT1aN0	PR
PR4	60	Infiltrating carcinoma	cT4N1	IIIB	<1	0	3+	Left mastectomy and lymphadenectomy	Multiple foci of infiltrating carcinoma NST	yT1aN1	PR
PR5	53	Infiltrating carcinoma	cT4N0	IIIB	0	0	3+	Right mastectomy and lymphadenectomy	Foci of ductal infiltrating carcinoma	yT1aN0	PR
PR6	45	Infiltrating carcinoma	cT4N0	IIIB	0	5	3+	Left radical mastectomy and axillar lymphadenectomy	Multiple foci of DCIS and dermal infiltration of carcinoma	yTisN2	PR

### Sample Extraction and Preparation

All the samples were checked with H&E by a senior pathologist who confirmed the low presence of stromal cells in favor of tumor cells, surely over the 90%.

Genomic DNA was extracted from four 5 μm sections of FFPE primary tumor or from ten 5 μm sections of FFPE tumor biopsies of each sample using the Maxwell® 16 FFPE Tissue LEV DNA Purification Kit (Promega, Madison, WI). DNA samples were then amplified using GenomePlex® Single Cell Whole Genome Amplification Kit (Sigma-Aldrich, Saint Louis, MO).

### Library Preparation and Whole-Exome Analysis

Whole-exome library preparation was performed using Ion TargetSeq™ Exome Enrichment Kit (Thermo Fisher, Whaltam, MA) and the Nextera Rapid Capture Expanded Exome Kit (Illumina, San Diego, California, U.S.) following manufacturer procedure. Exome analysis was performed using both Ion Proton™ Sequencer (Ion Torrent) and NextSeq™ 500 (Illumina, San Diego, California, U.S.).

### Bioinformatic Analysis

Data were automatically analyzed by using the Ion Torrent server, previously set for the alignment to the human genome (hg19 version). Raw data generated from Illumina NextSeq500™ were converted using Bcl2Fastq tools provided by Illumina. The primary Illumina data analysis of exomes was performed by using the SeqMule pipeline (25). VCF files obtained from exome analysis were filtered using Enlis Genome Research. We started using the following filter: quality score ≥10, read depth ≥30, allele frequency (as 1000 Genome Project and Exome Aggregation Consortium) <1% and protein impact involving missense, non-sense, frameshift and splice disrupt mutations. For missense mutations we used the Dann Model (26) to select the predicted deleterious alterations. A this point we have further refined the research by filtering the sample using specific database as COSMIC Database, HerceptinR: Herceptin Resistance Database (http://crdd.osdd.net/raghava/herceptinr/index.html) and a custom list of predicted driver genes obtained from CRAVAT (http://www.cravat.us/CRAVAT/), a web tool dedicated to discover driver mutations.

### Discriminant Analysis

A discriminant analysis was performed to predict the TRA resistance by mutational state. As independent variables, we considered the presence/absence of mutations in our list of 18 genes. The analysis was executed by using Tanagra software (https://eric.univ-lyon2.fr/ricco/tanagra/en/tanagra.html). A cluster analysis was also performed with the same genes by using Stata 12 (StataCorp LP).

### Cell Culture

Human breast cancer cell lines BT474 (ATCC® HTB-20™) deriving from a human breast ductal invasive carcinoma, were grown in DMEM with 10% fetal bovine serum (FBS), 100 U/mL penicillin/streptomycin, 0.01 g/L Insulin and 2 g/L HEPES. Cell lines were incubated at 37°C in a humidified atmosphere incubator containing 5% CO_2_.

### ANKRD44-shRNA Plasmid Silencing

SureSilencing shRNA Plasmid (Qiagen, Hilden, GE) was used for silencing the ANKRD44 gene. 8 × 10^4^ of BT474 cells were seeded in a 6-well plate and transfected in triplicate with Negative Control shRNA plasmid (shCTRL cells) and shRNA-ANKRD44 plasmid (shANK cells) following manufacturer procedure. Cells were then positively selected with 800 μg/ml of Geneticin, G418 (Sigma-Aldrich), and subsequently kept at a 350 μg/ml dose to maintain the gene silencing selection.

### Real Time PCR

Total RNA was extracted using the Maxwell® 16 LEV simplyRNA Cells Kit (Promega). Total RNA was then reverse transcribed to total cDNA using the nanoScript 2 Reverse Transcription kit (PrimerDesign Ltd., Eastleigh, UK) according to the manufacturer instructions. Real-time PCR was performed on cDNAs to detect relative expression of ANKRD44 and GAPDH for a loading control.

### Western Blot Analysis

For total protein extraction, cells were lysed in RIPA lysis buffer (SDS, Sigma-Aldrich), containing protease/phosphatase inhibitor cocktail (Cell Signaling Technology, Danvers, MA) following manufacturer procedure. 10–20 μg of proteins were separated on a 10% Mini-PROTEAN® TGX Stain-Free™ Precast Gels (Bio-Rad Laboratories) and electro transferred to nitrocellulose membranes (Trans-Blot^®^ Turbo™ Mini Nitrocellulose, Bio-Rad Laboratories). Membranes were then blocked with 5% BSA (Sigma-Aldrich) in PBS-Tween-20 (PBS-T) buffer and incubated overnight at 4°C with the following primary antibodies 1:350 rabbit polyclonal anti-ANKRD44 (Sigma-Aldrich), 1:1,000 rabbit polyclonal anti-Akt (Cell Signaling Technology), 1:1,000 rabbit polyclonal anti-phospho-Akt (Cell Signaling Technology), 1:200 rabbit polyclonal phospho-TAK1 antibody (Biorbyt Ltd., Cambridge, UK) and 1:1,000 rabbit polyclonal anti-beta Tubulin Antibody (Santa-Cruz Biotechnology, Santa Cruz, CA) used as loading control. Membranes were washed and incubated with the appropriate secondary antibody (Abcam, Cambridge, UK) for 1 h at room temperature. Blots were washed and hybridization signals were measured with ChemiDoc XRS System (Bio-Rad Laboratories). Images were analyzed quantitatively using ImageLab™ 4.1 Software (Bio-Rad Laboratories).

### Determination of p65-NF-κB Activity by ELISA

Proteins were extracted using Nuclear Extraction Kit (Abcam) according to manufacturer procedure and concentrations were determined by Bradford reagent (Sigma-Aldrich). NF-kβ p65 Transcription Factor Assay Kit (Abcam) was used for measure the p65-RELA transcription factor DNA binding activity. A known concentration of nuclear and cytoplasmic proteins were loaded into a 96-well containing a double stranded DNA (dsDNA) sequence highly specific for the NF-kβ response element immobilized onto the bottom of wells according to manufacturer procedure. The activity of NF-kβ (p65) was then detected by measuring absorbance values at 450 nm with an Infinite® 200 PRO NanoQuant plate reader (Tecan, Mannedorf, Switzerland). Values were then normalized on the respective amount of loaded protein.

### Cell Viability Assay-WST1

shCTRL and shANK cells (7 × 10^4^) were seeded in octuplicate into a 96-well plate, and allowed to adhere overnight in a humidified atmosphere of 5% CO_2_. Cells were treated with and without 21 μg/mL of Trastuzumab (Herceptin® 150 mg, Roche, Basel, SW) at 37°C for 48 h, incubated with WST-1 reagent (Clontech Laboratories, Mountain View, CA) for 1 h at 37°C and read at 450 nm with the Infinite® 200 PRO NanoQuant plate reader (Tecan) to assess viability. Values were then normalized on the respective untreated cells.

### Soft Agar Colony-Forming Assay

A 6-well plate was prepared with a base layer of 0.6% low melting point agarose, then 1 × 10^4^ of shCTRL and shANK cells were mixed with growth medium containing 0.4% agarose and then plated in triplicate onto each well and allow to solidify. Fresh media with and without TRA (21 μg/mL) was added on top and replaced every 72 h. Plates were incubated for 1 month in a humidified incubator and colonies were than fixed with 4% Paraformaldehyde and stained with 0.005% Crystal Violet (Sigma-Aldrich). Colonies bigger than 100 μm were counted manually in five randomly selected fields.

### Cell Cycle Analysis

Cells were seeded into a 6-well plate. After attachment to plastic surface, cells were treated with and without TRA (21 μg/mL) for 48 h then trypsinized and fixed for 30 min at 4°C with 70% ice cold Ethanol. 2 × 10^5^ cells were collected in a FACS tube, washed twice with PBS, and suspended in a 50 μg/mL Propidium Iodide (Sigma-Aldrich) and 20 μg/ml RNase (Sigma-Aldrich) solution. After 30 min of incubation at R.T. in the dark, cells were then scanned for fluorescence intensity in FL3 channel and analyzed with CyFlow® Cube 8 flow cytometer (Sysmex-Partec, Görlitz, GE). Analysis of flow cytometry data was carried out with FCS-Express 4 software (De Novo Software, Los Angeles, CA).

### Cytologic Analysis

To observe the morphology of both shANK and shCTRL cells, we spread a drop from each cell suspensions over the glass-slide. Then the slides were allowed to air dry for 30 min and fixed for 10 min with 4% formaldehyde. At the end the slides were stained with Papanicolaou stain and reviewed by a senior cytopathologist under the inverted microscope Carl Zeiss Axio Observer Z1FLMot (Carl Zeiss Microscopy GmbH, Oberkochen, GE) who evaluated the main characteristics of malignancy in shANK cells: nuclear pleomorphism, ratio, multinucleation, pleomorphism of nucleoli and presence of multiple nucleoli. Images were taken with Carl Zeiss AxioCam Icc1 camera (Carl Zeiss Microscopy GmbH). These data were then confirmed through flow cytometric analysis, where multinucleated cells were detected with a propidium iodide staining. Analysis of flow cytometry data was carried out with FCS-Express 4 software (De Novo Software).

### Quantitative Proteome Analysis by LC-MS/MS

shCTRL and shANK cells were both treated and untreated with TRA (21 μg/mL) for 48 h. The LC-MS/MS experiments were performed as described previously (27). Briefly, 2 μg of proteins for each sample were digested with the SP3 digestion protocol (28) and the resulting peptides labeled with Tandem Mass Tag 10-plex (Thermo Fisher, Waltham, MA, USA). Labeled peptides were loaded on to RPS cartridges for high-pH fractionation with the AssayMap BRAVO platform (Agilent Technologies, Santa Clara, CA).

Samples were injected into an EASY-nLC 1000 coupled to an Orbitrap Fusion (Thermo Fisher) and analyzed with a data-dependent method with multi-notch synchronous precursor selection and MS3 scanning for TMT quantification.

Raw data files were analyzed using Proteome Discoverer 2.1 (Thermo Fisher). MS2 spectra were searched using SequestHT against a Swissprot Homo sapiens database (20,066 sequences) supplemented with a common contaminant database (246 sequences). Searches were performed using the TMT reagents (+229.163 Da, lysine and N-termini) and carbamidomethyl (+57.021 Da, cysteine) as static modifications, methionine oxidation (+15.995 Da) as dynamic modification, 20 ppm precursor mass tolerance, 0.6 Da fragment mass tolerance, and 20 ppm reporter ions tolerance. The search was performed using fully tryptic peptides with a minimum length of 6 amino acids and up to 2 missed cleavages. Results were filtered for a 1% false discovery rate (FDR) using the Percolator algorithm and additionally filtered for a minimum Xcorr of 1.8. Statistical analysis was performed with Perseus software.

### Droplet Digital PCR (ddPCR)

shCTRL and shANK cells were both treated and untreated with TRA (21 μg/ml) for 48 h. Total RNA was extracted and reverse transcribed as described above. ddPCR was performed on QX100™ ddPCR (Bio-Rad Laboratories) to detect relative expression of LDHB and TACSTD2 using GAPDH as loading control. Fluorescence signal quantification was performed by the droplet reader and the QuantaSoft™ software (Bio-Rad Laboratories). The ratio of positive vs. negative droplets was used to determine the number of mRNA copies per ml of the target molecule in the input reaction. Triplicate ddPCR analyses were performed per sample.

### Seahorse XFp Cell Energy Phenotype Test

Metabolic analyses were performed in the Agilent Seahorse XFp Analyzer (Agilent Technologies). This instrument creates a transient micro-chamber of only a few microliters in specialized cell culture microplates which enables the oxygen consumption rate (OCR), and the extracellular acidification rate (ECAR), to be monitored in real time. shCTRL and shANK cells were seeded into a 6-well plate and incubated overnight in a humidified CO_2_ incubator. Medium was then removed and replaced by fresh culture media with and without TRA (21 μg/mL). After 48 h of drug treatment, cells were plated in 8-well plates for 24 h (2 × 10^5^ cells per well) in complete media. Cells were changed to unbuffered DMEM (DMEM supplemented either 25 mM glucose, 1 mM sodium pyruvate, 31 mM NaCl, 2 mM GlutaMax, pH 7.4) and incubated at 37°C in a non-CO_2_ incubator for 60 min. Three measurements were taken before and after sequential injection of mitochondrial inhibitors (1.0 μM oligomycin and 1.0 μM FCCP). OCR and ECAR were automatically calculated by Seahorse XFp software. Every point represents an average of *n* = 3.

### ROS Production Assay

Reactive oxygen species (ROS) production was quantitated using CellROX® Oxidative Stress Reagents (Thermo Fisher) a fluorogenic probe, according to the manufacturer instructions. 7 × 10^4^ shCTRL and shANK cells were seeded in octuplicate into a 96-well plate, and the plate was incubated for 24 h. Cells were incubated for another 48 h with and without TRA (21 μg/mL), then 100 μL of normal media and CellROX reagent (2.5 mM) were added in each well and plates were incubated for 1 h at 37°C. Green fluorescence values at 520 nm were measured with the Infinite® 200 PRO NanoQuant plate reader (Tecan). Values were then normalized on the respective untreated cells.

### Statistical Analysis

Experiments were repeated independently a minimum of three times and values were expressed as means–standard deviation (SD). Values of *p* < 0.05, *p* < 0.01, or *p* < 0.001 where used, indicate statistical significance.

## Results

### Whole-Exome Analysis Results

**Figure 1:**
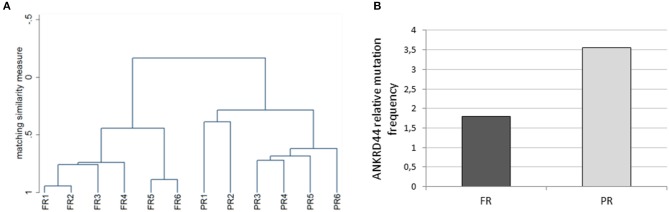
Whole exome analysis. **(A)** The dendrogram derived from cluster analysis classified 12 Her2 breast cancer patients based on the mutational profile of 18 informative genes. **(B)** Histogram representing the ANKRD44 gene relative mutational frequency between full (FR) and partial (PR) responder patients. There is a 2-fold higher mutation frequency in the PR than in the FR samples. Values are normalized on the total amount of mutations found per group (FR and PR).

We performed whole exome analysis of 12 Her2-like BC patients treated with a TRA neoadjuvant based therapy. They were classified on the basis of the response to treatment, in 6 FR and 6 PR patients. Through NGS analysis, we identified a total of 18 informative gene mutations present in the FR or PR patients: OR6C74, CEP350, ANKRD44, RPTN, FAM161A, FAM175A, MAPK1, FOLH1, GMPR, TGM1, ITPR1, LCT, PIK3C2G, NCBP1, OR10J5, FAT1, MUC16, and LRG1 (Table 2). We performed discriminant analysis to determine whether the presence of mutations in the 18 genes discriminate between FR and PR patients. This is remarked graphically also by the dendrogram (Figure 1A) performed after hierarchical clustering analysis.

**Table 2:** Whole exome analysis: list of the 18 informative gene mutations and corresponding aminoacid changes.

**Gene**	**Description**	**rs ID**	**A.A. change**
OR6C74	Olfactory receptor family 6 subfamily C member 74	rs4522268	R-62-X-
CEP350	Centrosomal protein 350	rs12124336	S-1517-A
ANKRD44	Ankyrin repeat domain 44	rs35338671	I-94-M
RPTN	Repetin	rs75957773	E-707-G
FAM161A	Family with sequence similarity 161 member A	rs17513722	I-236-V
FAM175A	Abraxas 1, BRCA1 A complex subunit	rs12642536	A-348-U
MAPK1	Mitogen-activated protein kinase 1	rs6928	3′-UTR
FOLH1	Folate hydrolase 1	rs75940285	R-175-W
GMPR	Guanosine monophosphate reductase	rs747618542	A-274-T
TGM1	Transglutaminase 1	rs549195122	R-225-H
ITPR1	Inositol 1,4,5-trisphosphate receptor type 1	–	E-2648-K
LCT	Lactase	–	R-509-H
PIK3C2G	Phosphatidylinositol-4-phosphate 3-kinase catalytic subunit type 2 gamma	rs12312266	P-977-L
NCBP1	Nuclear cap binding protein subunit 1	–	R-401-C
OR10J5	Olfactory receptor family 10 subfamily J member 5	–	P-182-L
FAT1	FAT atypical cadherin 1	rs751999701	R-2569-H
MUC16	Mucin 16, cell surface associated	rs200813849	N-13594-D
LRG1	Leucine rich alpha-2-glycoprotein 1	rs966384	P-133-S

*Empty cells (–) are mutations not yet discovered*.

Interestingly, 50% of PR patients (3 of 6) presents a particular ANKRD44 SNP (Table 2—rs35338671) predicted to be deleterious which leads to a I94M amino acid exchange. In order to evaluate the effect of this variant on ANKRD44 protein, we perform an *in silico* test by using MutationTaster software (http://www.mutationtaster.org/). As expected, the rs35338671 SNP (Table 2) has an high probability to be deleterious for protein conformation that leads to loss of the 3rd repeat domain of the ANKRD44 protein thus interfering with the structure and stability of the protein itself. Moreover, through an in-depth mutational profile analysis of the two groups of patients we found that ANKRD44 gene presents a higher number of deleterious mutations in the PR patients than in FR patients (Figure 1B). Furthermore, the expression values of ANKRD44 were evaluated for most patients, based on their availability. In general, lower expression was observed in PR patients compared to FR. The data are shown in Figure S1.

These data underline a potential link between ANKRD44 gene alteration and TRA resistance.

### ANKRD44 Silencing Confers Partial Trastuzumab Resistance and a More Aggressive Phenotype

**Figure 2:**
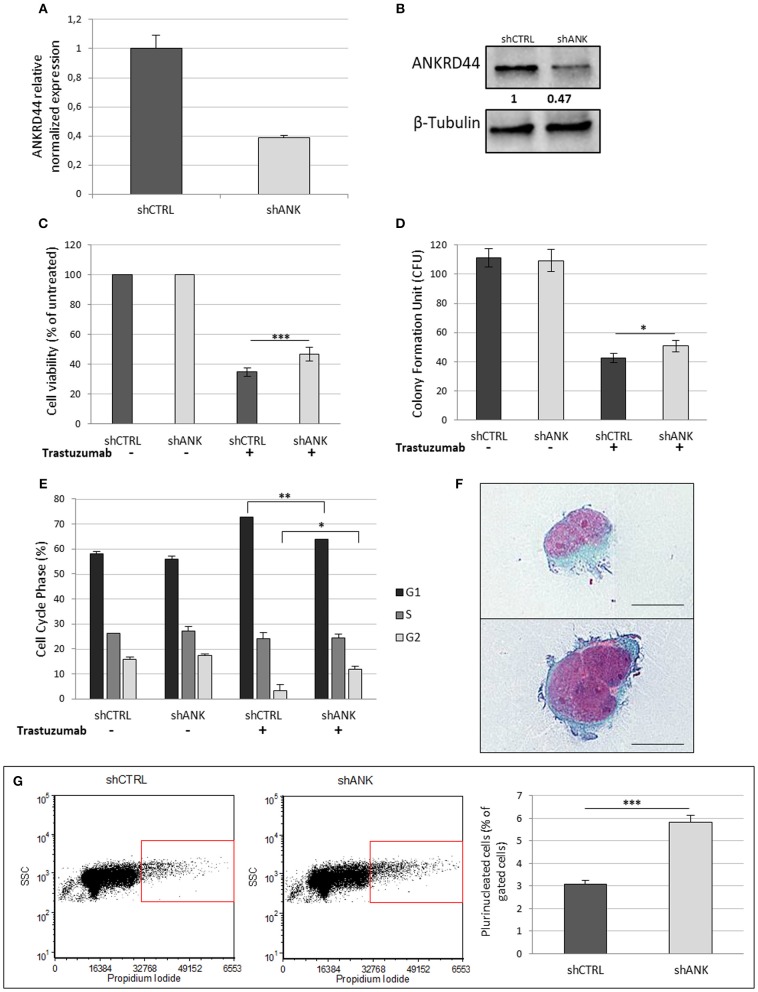
ANKRD44 silencing confers partial trastuzumab resistance and a more aggressive phenotype: **(A)** ANKRD44 mRNA expression detected by quantitative real-time RT-PCR analysis in BT474 cells with control empty vector (shCTRL) and with ANKRD44 short interfering RNA (shANK). **(A)** Gene expression values were normalized on housekeeping GAPDH mRNA. **(B)** Western Blot of proteins extracted from shANK and shCTRL cells. β-Tubulin was used as reference. The band intensity of ANKRD44 was normalized against the respective β-tubulin, and the ratios shown are against the shCTRL. Data were obtained with ImageLab™ 4.1 Software (Bio-Rad). **(C)** Viable cell count assessed by WST1 assay. Values were normalized on untreated cells. **(D)** Colony formation assay histogram. shANK cells present a 10% increase in the colony formation number respect to the shCTRL cells. Colonies larger than 100 μm in diameter were counted from five randomly selected fields. **(E)** Cell cycle distribution of shCTRL and shANK cells performed with propidium iodide staining in both untreated (–) and treated (+). Bars present the percent of cells in G1/G0-phase (black), in S phase (dark gray) and in G2/M phase (gray). **(F)** Two examples of plurinucleated cells with anisonucleosis obtained by cytochemistry analysis in shANK cells (Papanicolaou stain × 200). Scale bars: 25 μm. **(G)** Plurinucleate cells distribution of shANK (right) and shCTRL (left) cells fixed and labeled with propidium iodide in a FL3/SSC dot plot. Quadrant gate regions identify cells with higher scatter intensity (high SSC) and higher concentration of PI, index of cells with big dimensions, more intracellular complexity and nuclei dysmetria. Percentage of gated cells has been reported in a histogram and shANK cells present a significant (*p* < 0.01) increase of multinucleated cells respect to shCTRL cells. Each experiment was repeated in triplicate **(D,E)** and octuplicate **(C)** and each bar represents the mean ± SD of 3 independent experiments. Statistical significance was examined using Student *t*-test. (^*^p < 0.05; ^**^p < 0.01; ^***^p < 0.001).

Her2-like BT474 BC cell line were transfected with shRNA-ANKRD44 plasmid (shANK cells) and shRNA-Control plasmid (shCTRL cells). The expression levels of ANKRD44 gene in shANK cells and shCTRL cells were examined using real-time PCR and Western Blot. As shown in Figures 2A,B, ANKRD44 gene and protein was 2-fold downregulated in shANK cells respect to the shCTRL cells (*p* < 0.01).

#### Cell Viability

To determine whether ANKRD44 is involved in TRA resistance, we examined the effect of TRA on cell viability in both shCTRL and shANK cells. After 48 h of TRA treatment we observed a 13% increase in shANK proliferation (*p* < 0.001) respect to the shCTRL cells (Figure 2C). WST1 assays showed that shCTRL cell viability was reduced by 65% after TRA treatment while shANK cells were less affected by the same treatment (52%).

#### Colony Assay

A similar observation was also noted in the colony formation assay, which showed that the transformation potential of shANK cells is significantly higher than that of the shCTRL cells (*p* < 0.05; Figure 2D). These results suggest that the shANK cells present a more aggressive phenotype respect to the shCTRL cells.

#### Cell Cycle Analysis

The analysis of the cell cycle phases by flow cytometry (Figure 2E) showed that TRA induces an increase of the G1 phase (14.7%) in the shCTRL and a corresponding decrease (12.6%) of the proliferative cells in S/G2 phases indicating a block in G1 phase. Interestingly, shANK cells are less affected by the G1 arrest caused by TRA treatment respect to shCTRL cells. In fact, drug treatment in shANK cells causes a less accentuated increase in the G1 phase (7.9%) and a decrease in G2 (5.5%) with respect to the shCTRL cells (*p* < 0.01 and *p* < 0.05, respectively).

#### Cell Morphology Analysis

By a morphological comparison of both shANK cells and shCTRL cells after Papanicolaou staining, shANK cells present an increased number of multinucleated cells with nuclear dysmetria and anisonucleosis (Figure 2F). These features are typical of the de-differentiated cells that present a more aggressive cancer phenotype. Morphological data were also confirmed through flow cytometric analysis of shANK and shCTRL cells both stained with propidium iodide (Figure 2G). In fact, shANK cells present a statistically significant (*p* < 0.001) increase of multinucleated cells respect to the shCTRL cells.

### ANKRD44 Silencing Increase NF-κβ (p65) Activity Through TAK1/Akt-Pathway

**Figure 3:**
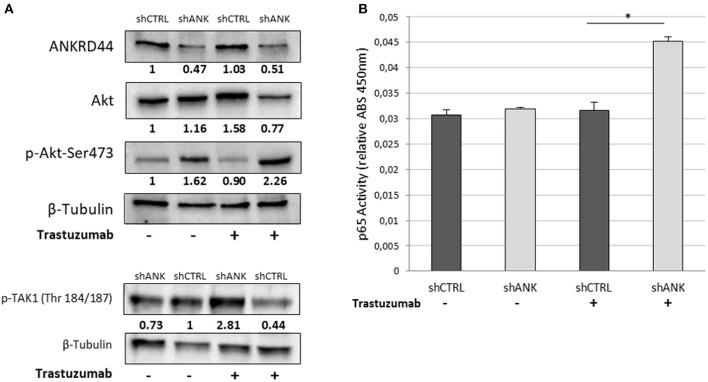
ANKRD44 silencing increases NF-kβ (p65) activity through TAK1/Akt-pathway. BT474 cells with empty vector (shCTRL) and with ANKRD44 shRNA (shANK). Cells were treated (+) and untreated (–) with trastuzumab 21 μg/ml for 48 h. **(A)** ANKRD44, Akt and p-Akt-Ser473 western blots. **(B)** Western blot of p-TAK1 (Thr 184/187). Proteins band intensity was normalized against the respective β-tubulin using ImageLab™ 4.1 Software (Bio-Rad) and normalized on untreated shCTRL cells. **(C)** NF-kβ (p65) activity of shANK cells and shCTRL cells. Values represent absorbance measurement (450 nm) with an Infinite 200 PRO NanoQuant plate reader (Tecan). Values were then normalized on the respective amount of loaded protein. Columns, mean of 3 determinations. Statistical significance was examined using Student *t*-test. (^*^p < 0.05).

We investigated whether there was any change in the expression and phosphorylation level of TAK1 and Akt kinases, which are, negatively regulated by ANKRD44 in physiological conditions (22, 23). Western blot analysis confirmed silencing of ANKRD44 (shANK) as shown in the first panel of the Figure 3A. Moreover, we observed that the total form of Akt decreases in shANK cells treated with TRA respect to the shCTRL. This result is in accordance with the significant increase of the phosphorylated forms of Akt (p-Akt-Ser473) and TAK1 (p-TAK1 Thr184/187) in shANK cells after treatment compared to the shCTRL (Figures 3A,B). Thus, the results demonstrate that silencing of ANKRD44 confers to shANK cells an over-activation of TAK1/Akt protein after TRA treatment. Furthermore, we extended our study to examine if this over activation of TAK1/Akt affects the activity of phosphorylated p65-NF-kβ in shANK cells. As shown in Figure 3C, ELISA results indicated that p65-NF-κβ levels were increased when shANK cells were treated with TRA (*p* < 0.05) respect to shCTRL cells. Thus, the data indicate that NF-kβ activation in shANK cells could be initiated by ANKRD44 silencing through the TAK1/Akt pathway.

### ANKRD44 Silencing Confers a Different Proteomic Pattern in shANK Cells Respect to the Control

**Figure 4:**
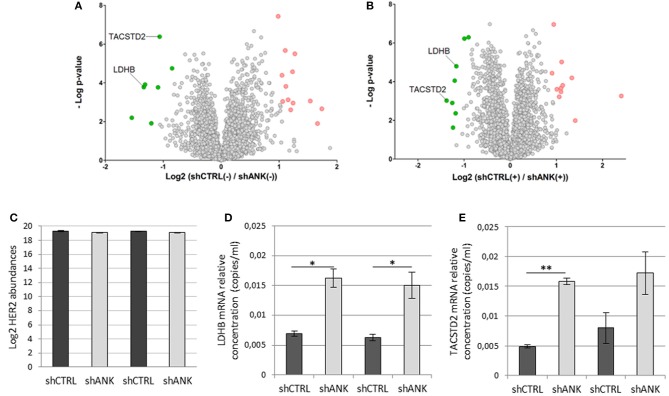
ANKRD44 silencing confers a different proteomic pattern in shANK cells respect to the control. Volcano plot showing the protein expression comparison between shCTRL and shANK cells before **(A)** and after **(B)** treatment with Trastuzumab. Gray dots represent protein not differently expressed (double sided *t*-test with FDR < 0.05). Green and red dots represent proteins significantly up-regulated and down-regulated in the shANK cells, respectively. **(C)** Log2 of the ERBB2 expression levels for the shCTRL and shANK before (–) and after (+) treatment with Trastuzumab. **(D,E)** LDHB and TACSTD2 mRNA expression detected by droplet digital PCR (ddPCR) analysis in BT474 cells with control empty vector (shCTRL) and with ANKRD44 short interfering RNA (shANK) both treated and untreated for 48 h with Trastuzumab 21 μg/ml. Gene expression values were normalized on housekeeping GAPDH mRNA and the values reported (copies per ml) represent the mean of the triplicate per sample. Bars: SD. Statistical significance was examined using Student *t*-test. (^*^p < 0.05; ^**^p < 0.01).

To identify possible changes in the proteome after ANKRD44 silencing, we performed quantitative proteomic profiling of shCTRL and shANK cells. A statistical analysis was performed to look for different protein expression profiles. To assess the significance of the protein expression changes we performed a two-sided Student's *t*-test using a permutation-based FDR cutoff of 0.05. Proteins were ranked in a volcano plot (Figures 4A,B) according to their statistical *p*-value (y-axis) and their fold change (log2 ratio of the protein abundances). As shown in Figure 4A, 7 proteins were up-regulated and 13 proteins were down-regulated in the untreated shANK cells compared to the control. In the treated cells (Figure 4B), 8 proteins were up-regulated and 11 proteins were down-regulated in the shANK cells compared to the shCTRL cells. The list of statistically significant proteins is reported in Table 3. Interestingly, the levels of HER-2 in shCTRL and shANK cells was not significantly different before and after treatment with TRA (Figure 4C) indicating that the effects observed are due to the ANKRD44 silencing alone. Among the different proteins obtained by the analysis, we focused our attention on LDHB and TACTSD2 found up-regulated in shANK cells both treated and untreated (Figures 4A,B). Through ddPCR we confirmed that also the mRNA levels of these two proteins were up-regulated in shANK cells respect to the shCTRL cells in both treated and untreated cells (Figures 4D,E).

**Table 3:** Proteome analysis: List of proteins with statistically significant differences in protein expression between shCTRL and shANK cells before and after treatment with Trastuzumab (double sided *t*-test with FDR < 0.05).

**Uniprot accession**	**Gene name**	**Fold change Log2(shCTRL (-)/shANK(-))**	**–Log(*p*-value)**
**shCTRL(−) vs. shANK(−)**			
O00339	MATN2	1.7	2.7
P29508	SERPINB3	1.7	1.9
P06753	TPM3	1.5	3.1
Q14956	GPNMB	1.3	5.5
P22455	FGFR4	1.2	3.0
Q5KU26	COLEC12	1.2	4.6
Q9UGT4	SUSD2	1.2	2.6
P31431	SDC4	1.2	3.1
Q9C0H2	TTYH3	1.1	3.8
P21741	MDK	1.1	5.7
P00533	EGFR	1.1	3.0
O00592	PODXL	1.1	4.4
P20061	TCN1	1.0	7.4
P20810	CAST	−0.8	4.8
P09758	TACSTD2	−1.1	6.4
Q8NBJ4	GOLM1	−1.1	3.8
P40199	CEACAM6	−1.2	1.9
P00918	CA2	−1.3	3.9
P07195	LDHB	−1.3	3.8
A1L170	C1orf226	−1.5	2.2
**shCTRL(+) vs. shANK(+)**			
Q9UGT4	SUSD2	2.4	3.3
Q9BXS4	TMEM59	1.4	2.0
Q02338	BDH1	1.3	4.2
Q9NR99	MXRA5	1.1	3.8
P21741	MDK	1.1	5.0
P10909	CLU	1.1	3.7
P06753	TPM3	1.1	3.5
O00592	PODXL	1.1	3.2
Q9C0H2	TTYH3	1.0	3.6
P20061	TCN1	0.9	7.0
Q5KU26	COLEC12	0.9	4.4
P53999	SUB1	−0.9	6.3
P20810	CAST	−1.0	6.2
P07195	LDHB	−1.2	4.8
Q9Y365	STARD10	−1.2	2.4
Q9UKY7	CDV3	−1.2	4.1
P25098	ADRBK1	−1.2	1.6
P35241	RDX	−1.3	2.9
P09758	TACSTD2	−1.4	3.0

*Uniprot accession number, gene name, fold change and FDR adjusted p-value are reported for each protein*.

### ANKRD44 Silencing Leads Cells Metabolism to a More Intense Glycolysis

**Figure 5:**
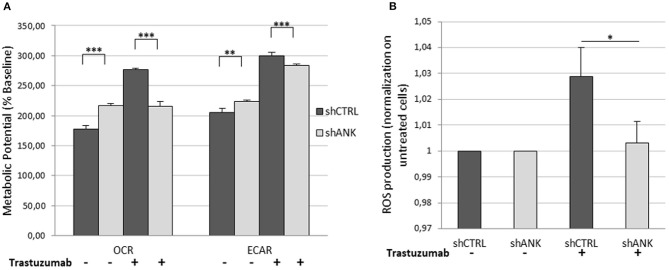
ANKRD44 silencing turn shANK (BT474) cells metabolism to a more intense glycolysis. BT474 cells with empty vector (shCTRL) and with ANKRD44 shRNA (shANK). Cells were treated (+) and untreated (–) with trastuzumab 21 μg/ml for 48 h. **(A)** Representative results of the metabolic potential of shCTRL and shANK cells measured using a Seahorse XF. Data shows OCR and ECAR, reflective of energy production by mitochondrial respiration and glycolysis, respectively, following sequential additions of oligomycin and FCCP. **(B)** Reactive oxygen species (ROS) production by shCTRL and shANK cells. Values represent green fluorescence measurement (520 nm) normalized on respective untreated cells. Each experiment was repeated in triplicate and each bar represents the mean ± SD of 3 independent experiments. Bars: SD. Statistical significance was examined using Student *t*-test. ^*^p < 0.05; ^**^p < 0.01; ^***^p < 0.001.

The LDHB protein up-regulation, obtained from LC-ms/ms analysis suggests that shANK cells could acquire a more intense glycolysis. To determine this hypothesis, we performed a metabolic potential analysis on shCTRL and shANK cells both treated and untreated with TRA. This analysis allows real-time measurements of ECAR and OCR, which are indicators of glycolysis and mitochondrial respiration, respectively. As shown in Figure 5A, we observed that untreated shANK cells presented higher glycolysis rate (ECAR) and mitochondrial activity (OCR) with respect to the shCTRL cells (*p* < 0.01). After TRA treatment, the trend is inverted and shCTRL cells have a significant higher ECAR and OCR compared to shANK cells. Interestingly, it is reported in literature that TRA treatment is associated with increased reactive oxygen species (ROS) production during mitochondrial respiration (29). Since ROS production increases H+ levels, which can interfere with ECAR probes, we decided to explore the possibility that the increased ECAR that we detected in the shCTRL cells could have been a false positive result due to ROS generation after TRA. Thus, we examined ROS generation in shCTRL and shANK cells after treatment using a commercial chemiluminescence assay kit. As shown in Figure 5B, the results indicate that ROS levels were increased when shCTRL cells were treated with TRA (*p* < 0.001) compared to shANK cells. So, the increase of ECAR in shCTRL cells is due to ROS production while in ANKRD44 silencing is associated to a real increase of glycolysis. This metabolic modification is consistent with the detected up-regulation of the LDHB protein in shANK cells.

## Discussion

Treatment of Her2-like BC patients is hindered by resistance to Her2-targeted therapies. Thus far, the mechanisms by which TRA inhibits Her2-positive BC development are still not fully defined. In the present study, through the comparison of patients with complete response and partial response to the neoadjuvant therapy with TRA, we identified 18 informative gene mutations that discriminate selectively the two groups of patients. Among the 18 genes, we focused our attention on ANKRD44 gene which mutation profile result to have a higher number of mutations in the PR compared to the FR patients. Moreover, the SNP rs35338671 identified in ANKRD44 gene leads to a loss of the 3rd repeat domain of the protein thus interfering with the structure and the stability of the protein. Interestingly, this particular SNP was present in 50% PR patients and absent in the FR patients. These results underlying not only the possible correlation between the 18 genes mutations and TRA treatment, but also an interesting correlation between ANKRD44 disruption and TRA resistance.

ANKRD44 gene encodes for an ankyrin repeat domain that, together with ANKRD28 and ANKRD52 proteins, create a putative regulatory ARS subunit of protein phosphatase 6 (PP6) complex (22, 23), a member of the PPP family of protein Ser/Thr phosphatases. The ARS subunit is involved in the recognition of phosphoprotein substrates that are subsequently dephosphorylated by the catalytic subunit PPP6C (24). Accumulating evidence shows that dysregulated PP6 activity plays a role in melanoma development (30), endometrial carcinoma (31) and hepatocellular carcinoma (32) but its role in BC is still unclear.

To understand if ANKRD44 gene could be involved in TRA resistance, we silenced this gene into a Her2-like human BC cell line (BT474). Actually, BT474 cells are Her2/ER/PgR positive, so they may behave differently than our patients who are Her2 positive but ER/PgR negative. Although, it must be considered the fact that TRA treatment is administered in both cases and for this reason we focused our attention only on the status of Her2.The expression levels of ANKRD44 gene and protein in both control (shCTRL) and silenced (shANK) cells were confirmed using real-time PCR and western blot analysis. Interestingly, shANK cells not only were less affected by TRA in the viability and in colony formation but also resulted to be less affected by the phase G1 arrest in the cell cycle caused usually by TRA treatment (33, 34) with respect to shCTRL cells. Furthermore, shANK cells showed particular nuclear features, typical of de-differentiated cells with a more aggressive cancer phenotype.

From these results we supposed that ANKRD44 gene could be involved in TRA resistance due to a miss-functional ARS subunit which is no longer able to detect phosphoprotein substrates for the catalytic subunit PPP6c. This impairment in PP6 activity may cause an increased activation of NF-kβ by TAK1/Akt pathway. In fact, in normal conditions PP6 blocks IL1 downstream signaling pathway (23, 35) by dephosphorylation of TAK1 (36). This kinase protein is no longer able to perform its function that leads to phosphorylation and activation of Akt (37), which in turn activate p38 and IKKS (38), implicated in the activation of p65-NF-kβ subunit (39), which enters the nucleus and allows transcription of genes involved in cell proliferation and in TRA resistance (20, 40). The activated form of TAK1 (pTAK-Thr184/187) and Akt (pAkt-Ser473) were found up-regulated in shANK cells after TRA treatment compared to shCTRL cells. These data confirm not only the strictly correlation between ANKRD44 silencing and TAK1 activation, but also the TRA resistance of shANK cells. Indeed, TRA treatment inhibits the PI3K/Akt pathway by Akt dephosphorylation (41, 42) as confirmed by shCTRL cells, which show a significantly decrease of pAkt when treated with TRA.

Since ANKRD44 silencing doesn't allow the inhibition of TAK1, the activation of the TAK1/Akt pathway in shANK cells results in increased NF-kβ activation (43) which does not occur in sensitive cell lines (shCTRL cells) with lower levels of pAkt. Indeed, we confirmed that p65-NF-kβ transcription factor was significantly activated in shANK cells after TRA treatment respect to the treated shCTRL cells despite Her2 expression was similar in both cell lines. The activation of NF-kβ induces downstream expression of inflammatory cytokines and anti-apoptotic proteins which promote cell proliferation (44). Interestingly, from LC-ms/ms analysis we found an up-regulation of TACSTD2, in shANK cells before and after treatment with TRA. Experiments conducted on human BC cell lines showed that TACSTD2 and NF-kβ have roles as regulators of gene expression with respect to one another (45). TACSTD2 expression is known to inhibit p27 (46), which can increase cyclin D/E levels associated with the transition from the G1 to the S phase and this finding is consistent with our results from the cell cycle analysis. As confirmation to our data, TACSTD2 is reported in the literature as associated to the epithelial-mesenchimal transition and therefore to a more aggressive profile, in BC (47).

As previously said, ANKRD44 silencing confers also a different proteomic pattern to the shANK cells with respect to control cells. In fact, we found several proteins that were up-regulated only in shANK cells before and after treatment, including LDHB. In addition, we found that RPTOR, GATA3, RPS6KB1, and RICTOR, which are proteins related to the mTOR pathway, were expressed only in shANK cells and not in shCTR cells (RPTOR has been quantified in at least three shANK replicates and not quantified in at least two shCTRL replicates). Interestingly, the activation of the mTOR pathway and the overexpression of LDHB has already been described in the literature (48) and could be associated with an impairment of PP6 activity due to ANKRD44 silencing. In fact, PP6 activity is strictly associated with mTOR signaling pathway by regulating ZNRF2 and eIF2α factors, regulators of mTORC1 activity (49, 50). LDHB is extensively described in the literature for its role in promoting tumorigenesis in colorectal cancer (51) and also for its involvement in the metastatic profile (52). Recently Nagamine et al. (53) underlined the connection between drug sensitivity and LDHB, which is overexpressed in cetuximab-resistant colorectal cancer cell lines. These data are consistent with the higher glycolysis rate that we observed in the shANK cells compared to shCTRL cells by metabolic potential measurements and it is also in accordance with the association found in the literature between a glycolytic phenotype and the TRA resistance (54, 55).

It is interesting to point out that the shCTRL cells appeared initially to have an increased glycolysis after treatment, proven to be due to ROS production. These data are in accordance with the generally accepted model that TRA-mediated growth inhibition occurs also through increase in ROS production (29).

**Figure 6:**
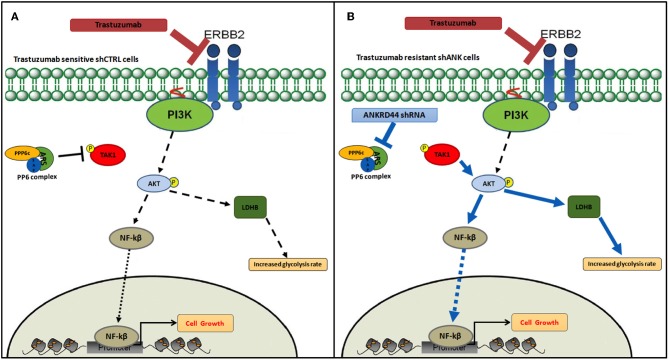
Snapshot of trastuzumab resistance pathway. Model of trastuzumab resistance acquisition in BT474 Her2+ breast cancer cell line due to ANKRD44 silencing. **(A)** In sensitive BT474 cells (shCTRL) without ANKRD44 silencing, trastuzumab blocks the downstream PI3K/Akt pathway (black dotted arrows) and prevents cell growth. **(B)** Thanks to ANKRD44 silencing BT474 cells (shANK) become partially resistant to the treatment due to an up-regulation of TAK1/Akt proteins which constitutively activate NF-kβ p65 subunit and leads to its nuclear accumulation (blue arrows). The nuclear p65 subunit activates the expression of target pro-inflammatory genes, survival genes and cell cycle regulators. Moreover, the TAK/Akt pathway activates mTORC1 complex which mediates cell proliferation and simultaneously increase glycolysis rate through an overexpression of Lactate dehydrogenase Beta protein (LDHB).

Based on all the results, in Figure 6, according to our results, we recapitulate TRA resistance in BC due to ANKRD44 silencing. In Figure 6A the mechanism of action of TRA in BT474 cells in normal conditions (shCTRL cells) is shown. TRA blocks the downstream PI3K/Akt pathway with consequent cell growth arrest. Instead, ANKRD44 silencing in shANK cells (Figure 6B) allows a partial acquisition of resistance to TRA due to the up-regulation of TAK1/Akt proteins which constitutively activates the NF-kβ p65 subunit and leads to its nuclear accumulation. NF-kβ p65 subunit in turn activates target pro-inflammatory genes, survival genes and cell cycle regulators. Indeed TAK1/Akt pathway activates LDHB with consequent increase in the glycolysis rate.

## Conclusions

In summary, through the comparison of the mutation profile of patients with different response to TRA treatment we identified ANKRD44 gene as a possible gene involved in TRA resistance. We have shown that ANKRD44 gene silencing in a Her2-like BC cell line activates NF-kβ through the TAK1/Akt pathway and thus induces a partially resistance to TRA treatment. Moreover, ANKRD44 silencing promotes an increased glycolysis rate in resistant cells confirmed by the up-regulation of LDHB protein. We showed that, after silencing, there is an increase in TACSTD2 protein expression which might be involved in increasing the transition from G1 to S phase despite TRA treatment. All of these results allow us to create a snapshot of the acquisition of TRA resistance in shANK cells after silencing ANKRD44 gene.

Together these results identify ANKRD44 gene as a novel key factor for TRA resistance but also as a possible predictive marker for the response to the neoadjuvant therapy with TRA. Moreover, it supports NF-kβ inhibition as a strategy for improving cell sensitivity to TRA.

## Data Availability

All data generated or analyzed during this study are included in this published article. The LC-MS/MS datasets generated and analyzed during the current study are available from the corresponding author on reasonable request.

## Ethics Statement

We confirmed that the study received ethical approval from the ethics committee of the Azienda Ospedaliero Universitaria Pisana (AOUP) with the EudraCT number 2018-001349-15 registrated in date 10/09/2014. All methods and experimental protocols were performed in accordance with the respective guidelines and regulations. All patients who participated in this study provided informed consent.

## Author Contributions

CMM and AF ideated and coordinated the project. MLF and FL wrote the manuscript, conducted all the experiments, and developed the whole project. DP carried out LC-MS/MS analyses and analyzed the proteomic data. FL supervised the project. ET and SF participated in the experimental designing. PA performed NGS and statistical analysis. IM performed cytological analysis. MM performed FFPE tissue sectioning and glass slides preparation. AGN and CrS analyzed and provided FFPE tissues and clinical analysis. CD, LD, IB, and MR provided patients samples. ClS and PC conducted part of whole exome library preparations. LM supervised the proteomic experiments. JH and MDR supported data analysis and manuscript editing. All authors have read and approved the final version of the manuscript.

### Conflict of Interest Statement

The authors declare that the research was conducted in the absence of any commercial or financial relationships that could be construed as a potential conflict of interest.

## Supplementary Material

Figure S1 In **(A)** the expression values of ANKRD44 of the single available FR and PR samples are shown; in **(B)** the mean expression value of ANKRD44 of PR and FR patients is reported.
