# Erratum: ANKRD44 Gene Silencing: A Putative Role in Trastuzumab Resistance in Her2-Like Breast Cancer

**DOI:** 10.3389/fonc.2019.00709

**Published:** 2019-07-18

**Authors:** Frontiers Production Office

**Affiliations:** Frontiers Media SA, Lausanne, Switzerland

**Keywords:** Her2+ breast cancer, Trastuzumab resistance, ANKRD44, next generation sequencing, LC-MS/MS, gene silencing

Due to a production error, the affiliation for Antonio G. Naccarato was incorrectly given as the “Department of Cell and Developmental Biology, the Hebrew University of Jerusalem, Jerusalem, Israel” (affiliation 8). The correct affiliation is the “Department of Translational Research and New Technologies in Medicine and Surgery, University Hospital of Pisa, Pisa, Italy” (affiliation 10).

The publisher apologizes for this mistake. The original article has been updated.

