# Supplementary figures and images for: ANKRD44 Gene Silencing: A Putative Role in Trastuzumab Resistance in Her2-Like Breast Cancer
Source: Front Oncol. 2019 Jun 26;9:547. doi: 10.3389/fonc.2019.00547 (PMC6607964; doi:10.3389/fonc.2019.00547)

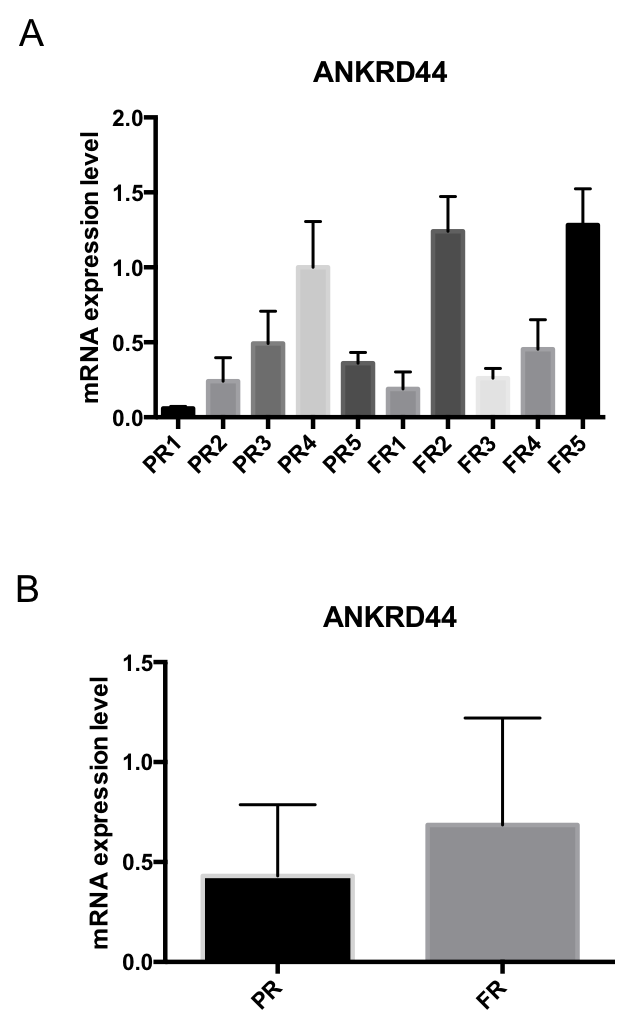

Supplement: Figure S1 — In (A) the expression values of ANKRD44 of the single available FR and PR samples are shown; in (B) the mean expression value of ANKRD44 of PR and FR patients is reported. [file Image_1.PNG]
